# Resveratrol protects osteocytes against oxidative stress in ovariectomized rats through AMPK/JNK1-dependent pathway leading to promotion of autophagy and inhibition of apoptosis

**DOI:** 10.1038/s41420-023-01331-2

**Published:** 2023-01-21

**Authors:** Liwei Wei, Shuang Chai, Chen Yue, Hong Zhang, Jitian Li, Na Qin

**Affiliations:** 1 Department of Sports Medicine, Luoyang Orthopedic-Traumatological Hospital (Orthopedics Hospital of Henan Province), Luoyang, Henan China; 2 Bone Pharmacology Laboratory, Luoyang Orthopedic-Traumatological Hospital (Orthopedics Hospital of Henan Province), Luoyang, Henan China

**Keywords:** Targeted bone remodelling, Target identification

## Abstract

A large number of studies in recent years indicate that osteocytes are the orchestrators of bone remodeling by regulating both osteoblast and osteoclast activities. Oxidative stress-induced osteocyte apoptosis plays critical roles in the pathological processes of postmenopausal osteoporosis. Resveratrol is a natural polyphenolic compound that ameliorates postmenopausal osteoporosis. However, whether resveratrol regulates osteocyte apoptosis via autophagy remains largely unknown. The effects of resveratrol on regulating osteocyte apoptosis and autophagy were analyzed both in vivo and in vitro. In vitro, cultured MLO-Y4 cells were exposed to H_2_O_2_ with or without resveratrol. In vivo, an ovariectomy-induced osteoporosis model was constructed in rats with or without daily intraperitoneal injection of 10 mg/kg body weight resveratrol. It was found that resveratrol attenuated H_2_O_2_-induced apoptosis through activating autophagy in cultured MLO-Y4 cells, which was mediated by the dissociation of Beclin-1/Bcl-2 complex in AMPK/JNK1-dependent pathway, ultimately regulating osteocytes function. Furthermore, it was shown that resveratrol treatment reduced osteocytes oxidative stress, inhibited osteocytes apoptosis and promoted autophagy in ovariectomized rats. Our study suggests that resveratrol protects against oxidative stress by restoring osteocytes autophagy and alleviating apoptosis via AMPK/JNK1 activation, therefore dissociating Bcl-2 from Beclin-1.

## Introduction

Postmenopausal osteoporosis is characterized by mechanical unloading and low estrogen levels due to a decrease in bone density and micro-architectural deterioration of bone tissues [1, 2]. Bone-forming osteoblasts and bone-resorbing osteoclasts, accompanied with osteocytes, are involved in the process of bone remodeling, which can be embedded within the mineralized matrix of the bones [3]. Numerous studies now show that osteocytes, as the most abundant cells in bone, can serve as the orchestrators of bone remolding by modulating the functions of osteoclasts and osteoblasts [4]. The viable osteocytes are capable to repair bone damage, maintain bone density, and ensure an efficient remodeling process [5, 6]. Thus, improving the viability of osteocytes may serve as a potential strategy for treating postmenopausal osteoporosis.

Growing evidence suggests that oxidative stress is increased with estrogen deficiency and aging, thus leading to the development of postmenopausal osteoporosis [7, 8]. ROS induces the apoptotic levels of osteocytes and osteoblasts, which suppresses osteogenesis or bone mineralization and promotes osteoclastogenesis [9]. Oxidative stress can induce apoptosis in osteocytes, which leads to an imbalance between bone formation by osteoblasts and bone resorption by osteoclasts and ultimately impairs bone remodeling and promotes bone loss [10]. Besides, the apoptotic osteocytes are involved in the recruitment of osteoclasts to the bone sites, thereby improving bone resorption [11]. Autophagy is a crucial evolutionarily conserved intracellular process that affects cell growth, differentiation, development, and homeostasis. It can degrade and recycle damaged or long-lived cellular organelles and proteins, as well as maintain osteocyte homeostasis [12]. Numerous adverse factors, such as hypoxia and ROS, can affect osteocyte survival via regulation of autophagy [13]. Previous evidence has demonstrated that multiple signaling pathways such as ROS/AMPK and ROS/JNK/c-Jun are involved in oxidative stress-induced autophagy and apoptosis in bone remodeling [14]. Additionally, the interaction between autophagic protein Beclin1 and the anti-apoptotic protein Bcl-2 represents an important link between autophagy and apoptosis [15]. MLO-Y4 cells after fluid shearing force exhibit AMPK signaling activation Using RNA-seq [16]. AMPK regulated RANKL and sclerostin expression through the mevalonate pathway in osteocytes [17]. However, the regulatory effect of autophagy mediated by AMPK/JNK1 signaling pathway on osteocyte apoptosis is less known.

Resveratrol is a naturally occurring antioxidant compound derived from grapes, peanuts, berries and white hellebore [18]. Previous data from our lab and from other investigators have suggested that resveratrol activates AMPK under different conditions [19–23]. Ayumu Takeno et al. demonstrated in vitro that AMPK activation protects against oxidative stress-induced apoptosis of osteocytes by regulating the expressions of NADPH oxidase 1 (Nox1) and Nox2 [24]. However, whether resveratrol modulates survival and function of osteocytes to inhibit postmenopausal osteoporosis is unknown and needs to be clearly elucidated. The present research aimed to assess whether resveratrol has a protective effect on postmenopausal osteoporosis via inducing osteocytes autophagy and inhibiting apoptosis. The target molecules involved in mediating these effects, such as AMPK and JNK1, were also assessed. We found resveratrol restored autophagy and inhibited apoptosis by disrupting the interaction between Beclin1 and Bcl-2 via AMPK/JNK1- mediated signaling pathways, thus contributing to the improvement of postmenopausal osteoporosis.

## Results

### Effects of resveratrol on osteocyte viability and oxidative stress

**Fig. 1:**
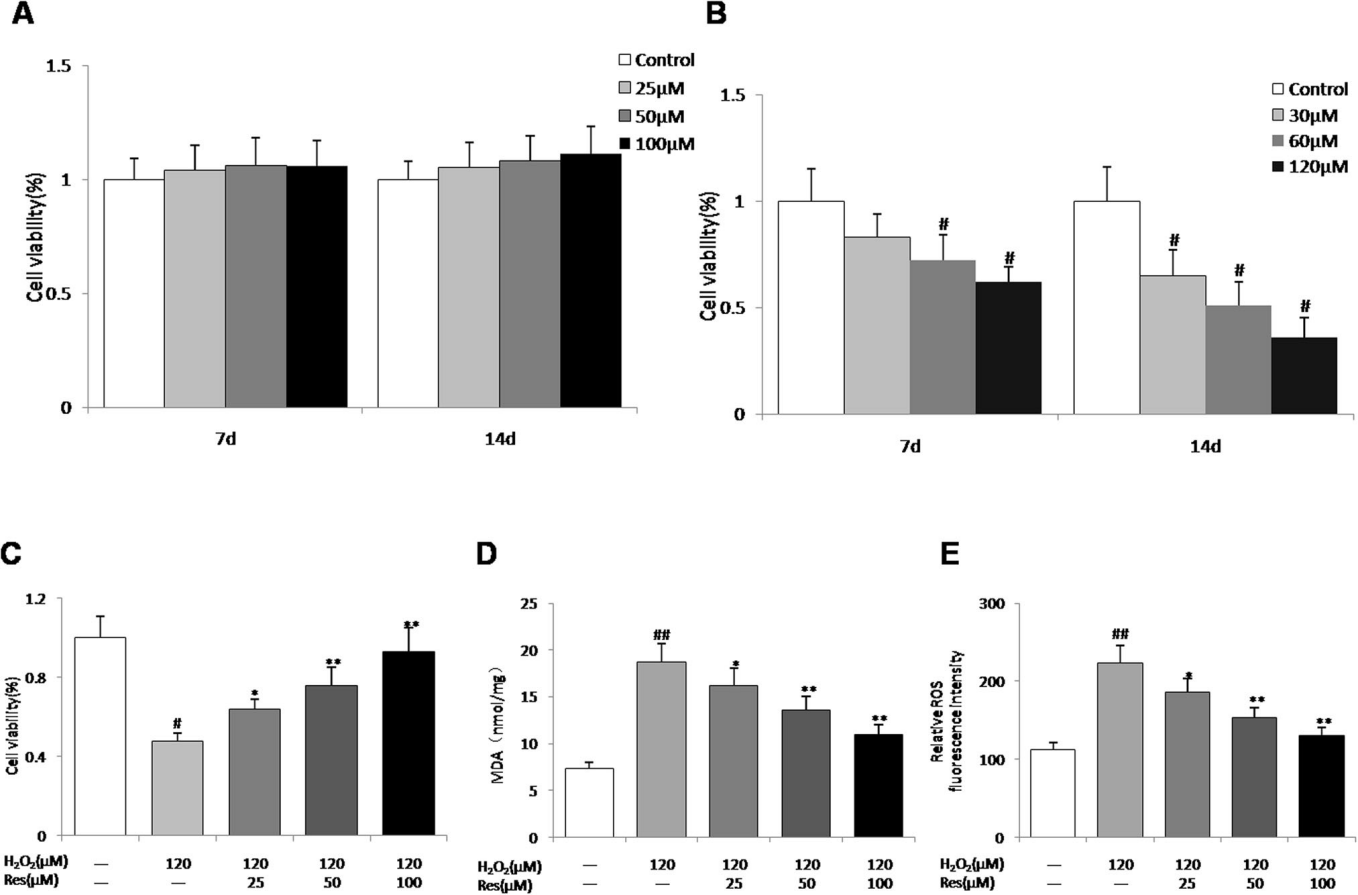
Efficacy of resveratrol on the viability of MLO-Y4 cells and oxidative stress. **A** Cell viability of MLO-Y4 cells following treatment with different concentrations of resveratrol from 7 d to 14 d. **B** Cell viability of MLO-Y4 cells in different concentrations of H_2_O_2_ from 7 d to 14 d. **C** Cell viability of MLO-Y4 cells administered with resveratrol for 24 h before 120 μM H_2_O_2_ administration for 14 d. **D**, **E** Effects of different concentrations of resveratrol on MDA and intracellular ROS content in MLO-Y4 cells supernatants after treatment with 120 μM H_2_O_2_. *n* = 6, ^#^*P* < 0.05 vs control, ^##^*P* < 0.01 vs control, *P < 0.05 vs H_2_O_2_, **P < 0.01 vs H_2_O_2_.

To assess whether resveratrol can affect the viability of osteocytes, MLO-Y4 cells were exposed to different doses (0, 30, 60, 120 μM) of H_2_O_2_ and pretreated with resveratrol, osteocyte viability was detected by using the CCK-8 assay. As shown in Fig. 1A, resveratrol had no significant effect on MLO-Y4 cell survival, but H_2_O_2_ reduced the viability of MLO-Y4 cells in a concentration- and time-dependent fashion, compared to the control group (Fig. 1B). When 25, 50, 100 μM resveratrol were exposed to MLO-Y4 cells for 24 hr prior to 120 μM H_2_O_2_ intervention for 14 d, viable MLO-Y4 cells significantly elevated compared to the untreated cells (Fig. 1C). To investigate whether the protective function of resveratrol is related to its antioxidant activity, the levels of MDA and ROS in MLO-Y4 cells were detected. Our results demonstrated that H_2_O_2_ increase oxidant activity and induced oxidative stress to MLO-Y4 cells. After pretreatment with resveratrol at various doses, the levels of MDA and ROS were dose-dependently inhibited (Fig. 1D, E). These findings indicated that resveratrol, in part, protected MLO-Y4 cells from H_2_O_2_-induced oxidative damage.

### Effects of resveratrol on osteocyte apoptosis and autophagy

**Fig. 2:**
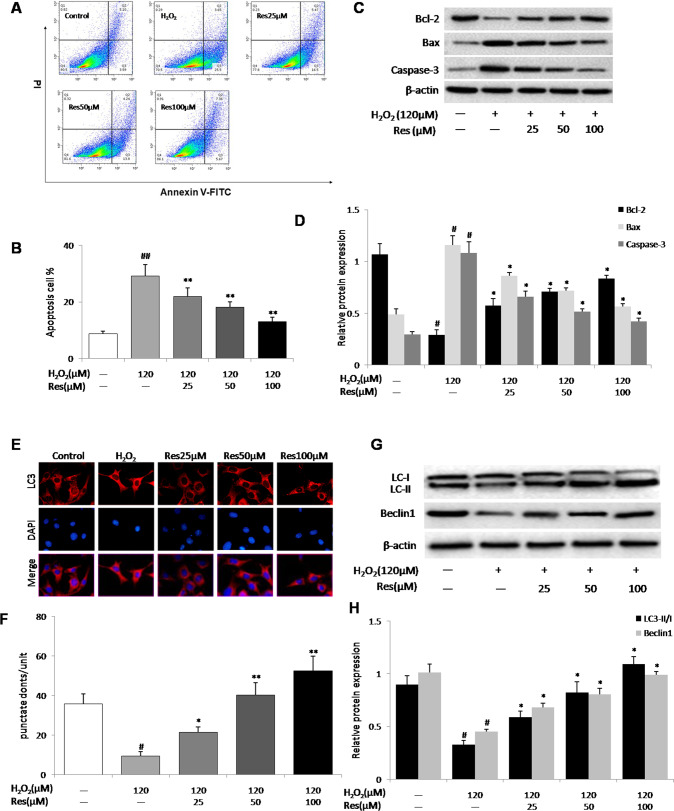
Resveratrol inhibits apoptosis and enhances autophagy induced by H_2_O_2_ in MLO-Y4 cells. Cells were exposed to H_2_O_2_ (120 μM) and treated with resveratrol (Res, 25, 50, 100 μM) for 14 d. **A** Representative diagram of annexin V/PI staining. **B** The apoptotic cells were calculated as the ratio of annexin V + /PI- cells to total cells. **C**, **D** The protein expression of Bcl-2, Bax and Caspase-3 in cell lysates were detected by western blotting. **E**, **F** MLO-Y4 cells were stained for LC3 (red arrows) and with the nuclear dye DAPI (blue) following the different treatments for 14 d. **G**, **H** The protein expression of LC3-II/I and Beclin1 in cell lysates were detected by western blotting. *n* = 6, ^#^*P* < 0.05 vs control, ^##^*P* < 0.01 vs control, *P < 0.05 vs H_2_O_2_, **P < 0.01 vs H_2_O_2_.

In order to evaluate the effect of resveratrol on H_2_O_2_-triggered MLO-Y4 cells apoptosis, FITC-Annexin V, PI staining and flow cytometric analysis were carried out. As demonstrated in Fig. 2A, B, the proportion of apoptotic MLO-Y4 cells treated with H_2_O_2_ 120 μM was 63.7 ± 8.0 %, which was remarkably higher than that of the untreated cells (*P* < 0.01). After pretreated with various concentrations of resveratrol (25-100 μM), the proportion of apoptotic MLO-Y4 cells were severely decreased. And the apoptotic cells were prominently decreased by 100 μM resveratrol compared with that of 25 μM resveratrol. Bcl-2/Bax ratio indicates the direction of apoptotic cells. Next, western blot analysis was used to evaluate the expression of Bcl-2 and Bax. Compared with the normal group, the ratio of Bcl-2/Bax in the H_2_O_2_ 120 μM group was significantly decreased. After resveratrol pretreatment, a ratio of Bcl-2/Bax elevated gradually. These results suggested that resveratrol could inhibit apoptosis in MLO-Y4 cells induced by H_2_O_2_ in a concentration-dependent fashion (Fig. 2C, D).

To explore whether resveratrol activates autophagy in MLO-Y4 cells, the intracellular level of the autophagy marker LC3 was detected by immunofluorescence. Our results showed that MLO-Y4 cells in the H_2_O_2_ 120 μM group had diffuse, weak cytoplasmic staining with red fluorescent protein (LC3), however MLO-Y4 cells pretreated with resveratrol showed increased fluorescence intensity, indicating that LC3-II is distributed within resveratrol-treated cells (Fig. 2E, F). To further confirm the autophagic role of resveratrol, the expressions of LC3-II, Beclin-1 were detected in MLO-Y4 cells of all groups by western blotting. Similarly, the H_2_O_2_-treated MLO-Y4 cells for 48 h expressed lower levels of LC3-II/LC3-I, Beclin-1 protein than that of the control group. Resveratrol treatment dose-dependently enhanced the expressions of LC3- II, Beclin-1 in MLO-Y4 cells (*P* < 0.01) (Fig. 2G, H).

### Resveratrol inhibits H_2_O_2_-trigerred apoptosis via activation of autophagy

**Fig. 3:**
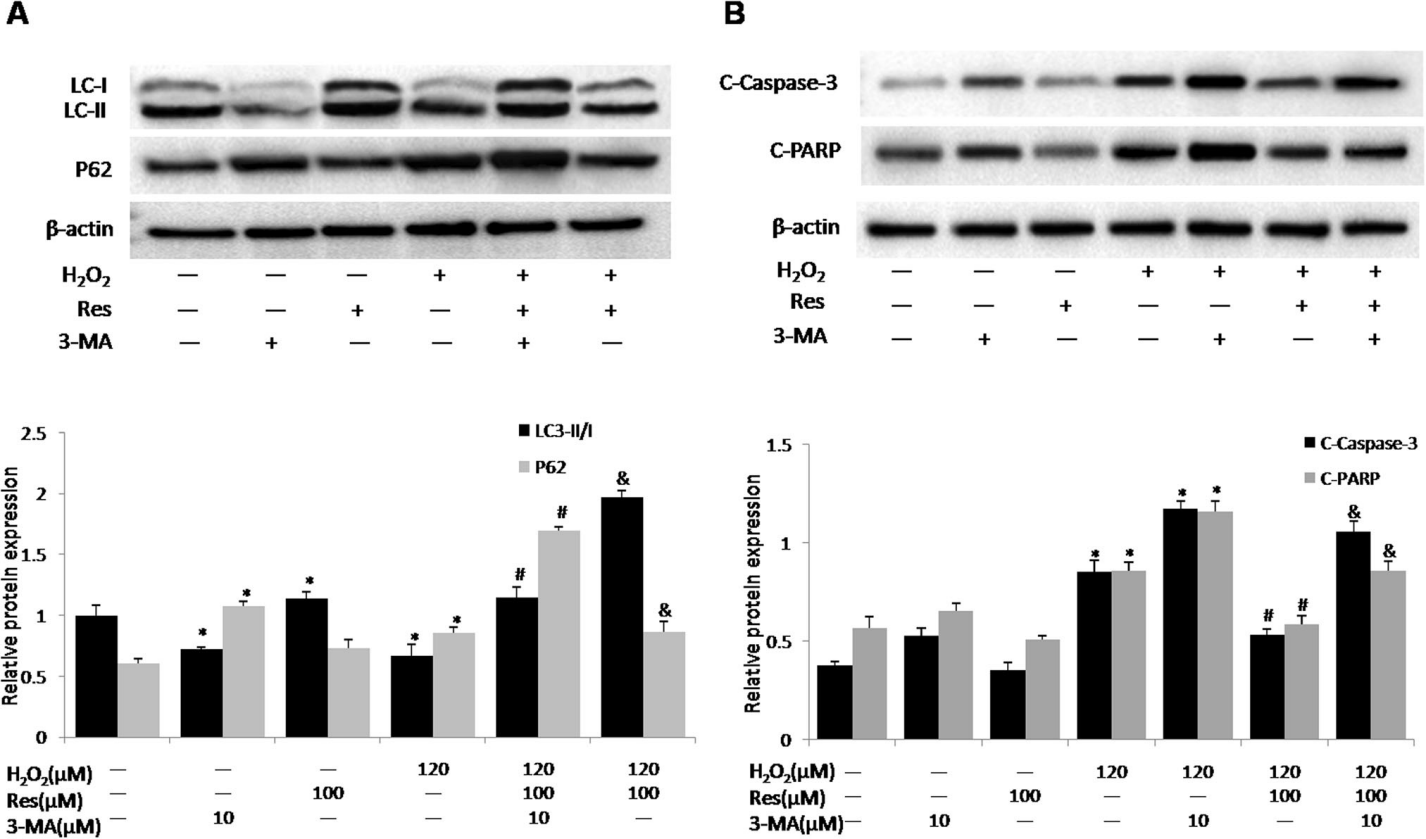
Resveratrol attenuates H_2_O_2_-induced apoptosis associated with enhanced autophagy. MLO-Y4 cells were pretreated with 3-methyladenine (3-MA, 10 μM) for 30 min before exposure to H_2_O_2_ (120 μM) and resveratrol (Res, 100 μM) for 14 d. **A** The protein expression of LC3-II/I and P62 in cell lysates were detected by western blotting. **B** Apoptosis markers including cleaved caspase-3 (C-caspase-3) and cleaved PARP (C-PARP) in cell lysates were detected by western blotting. *n* = 3; ^#^*P* < 0.05 vs. control, *P < 0.05 vs^.^ H_2_O_2_, ^&^*P* < 0.05 vs. H_2_O_2_/Res.

To examine the interactions between apoptosis and autophagy in resveratrol’ effect on MLO-Y4 cells, MLO-Y4 cells was exposed to the autophagy inhibitor 3-methylademine that mainly plays an inhibitory role in the formation of autophagosomes. Notably, resveratrol reversed H_2_O_2_-triggered decreases in P62-deposition and LC3- II production, which could offseted by adding 3-methylademine (Fig. 3A). Simultaneously, resveratrol reduced the levels of PARP and cleaved caspase-3 (the apoptotic markers) after exposure H_2_O_2_ to MLO-Y4 cells, which could be neutralized by 3-methylademine (Fig. 3B). This suggests that inhibition of autophagy can accelerate H_2_O_2_-triggered MLO-Y4 cell death, and the inhibition apoptosis of resveratrol is markedly associated with the activation of autophagy.

### Resveratrol activated AMPK/JNK1 to dissociate Bcl-2 and Beclin-1 in MLO-Y4 cells

**Fig. 4:**
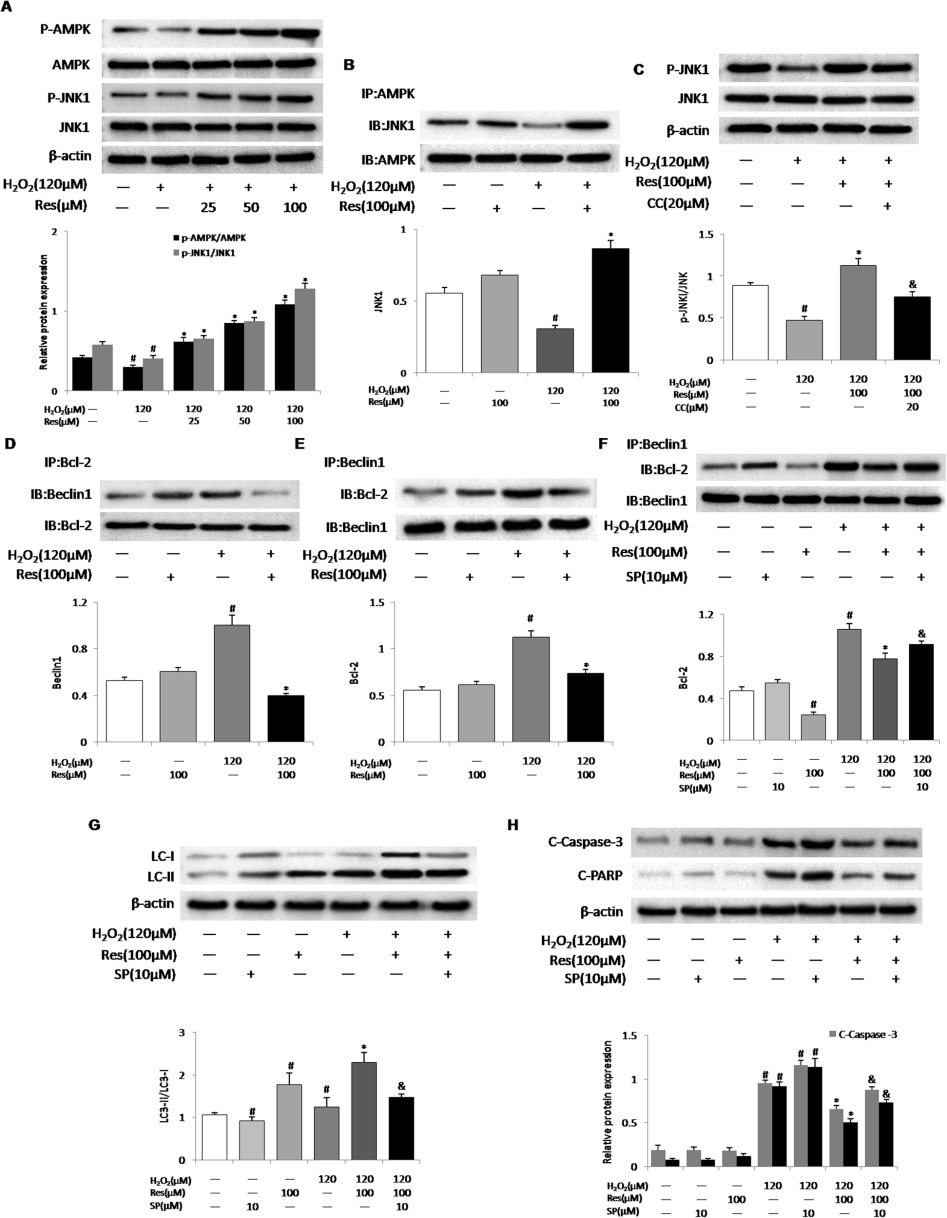
Resveratrol activates AMPK and JNK1 to disrupt the interactions between Beclin-1 and Bcl-2, thus promoting autophagy and inhibiting apoptosis. MLO-Y4 cells were pretreated with compound C (CC, 20 μM, AMPK inhibitor), or SP600125 (SP, 10 μM, JNK1 inhibitor) for 30 min before exposure to H_2_O_2_ (120 μM). MLO-Y4 Cells were treated with resveratrol for 14 d. **A** Resveratrol (Res, 25, 50, 100 μM) activated AMPK and JNK1 in MLO-Y4 cells exposed to H_2_O_2_. The levels of phosphorylated AMPKɑ (Thr172) and phosphorylation of JNK1 (Thr183/Tyr185) in cells lysates were measured by western blotting. **B** Resveratrol (Res, 100 μM) enhanced the interaction of AMPKɑ with JNK1 in MLO-Y4 cells exposed to H_2_O_2_. The association of AMPKɑ and JNK1 was assessed by immunoprecipitation (IP). **C** MLO-Y4 cells were pretreated with compound C (CC, 20 μM) for 30 min before exposure to 120 μM H_2_O_2_ and treated with resveratrol (Res, 100 μM) for 14 d, JNK1 and phosphorylation of JNK1 (Thr172) protein levels were measured by western blotting. **D**, **E** Beclin-1 or Bcl-2 was immunoprecipitated (IP) from cell lysates, and Bcl-2 or Beclin1 in the immunoprecipitate was detected by western blotting. **F** MLO-Y4 cells were pretreated with SP600125 (SP, 10 μM, JNK1 inhibitor) for 30 min before exposure to H_2_O_2_ (120 μM) and treated with resveratrol (Res, 100 μM) for 14 d, The association of Beclin-1 and Bcl-2 was assessed by immunoprecipitation (IP). **G**, **H** LC3-II/I protein levels and apoptosis markers (C-Casp3 and C-PARP) in cells lysates were measured by western blotting. *n* = 3; ^#^*P* < 0.05 vs. control, *P < 0.05 vs^.^ H_2_O_2_, ^&^*P* < 0.05 vs. H_2_O_2_/Res.

It has been reported that resveratrol can activate AMPK and JNK1 in a variety of cells [19–23]. Similarly, our results indicated that resveratrol dose-dependently elevated AMPK/JNK1 phosphorylation in MLO-Y4 cells (Fig. 4A). We first confirmed that AMPK and JNK1 were induced by resveratrol in H_2_O_2_-exposed MLO-Y4 cells. Moreover, the results of co-immunoprecipitation assays demonstrated that resveratrol remarkably enhanced the activation of AMPK/JNK1 after H_2_O_2_ intervention (Fig. 4B). Next, we determined if resveratrol caused JNK1 phosphorylation in AMPK-dependent manner. Compound C (CC), an AMPK inhibitor, can block the activation of AMPK. Consequently, reduced JNK1 phosphorylation was detected in CC-treated MLO-Y4 cells after exposure to H_2_O_2_ (Fig. 4C). These findings suggested both kinases could act coordinatively to regulate apoptosis and autophagy in resveratrol-treated MLO-Y4 cells.

Bcl-2 and Beclin-1 is a switch between apoptosis and autophagy [25, 26]. Research shows that activation of AMPK leads to phosphorylation of threonine 388 site of autophagy gene Beclin-1 [27], and the activated JNK1 may result in Bcl-2 phosphorylation [28, 29], which leads to dissociation of autophagy protein Beclin-1 and the members of the anti-apoptotic Bcl-2 family [30]. Immunoprecipitation assays showed that H_2_O_2_ strengthened the relationship between Bcl-2 and Beclin-1, and resveratrol could disrupt this interaction (Fig. 4D, E). Additionally, we used JNK1 inhibitors to further explore whether the action of resveratrol is related to the activation of JNK1. Our data suggested that the disruption of Beclin-1/Bcl-2 complex by resveratrol could be reversed by treatment with the JNK1 inhibitor SP600125 (Fig. 4F). Importantly, resveratrol-enhanced autophagy and inhibited apoptosis were attenuated by SP600125 treatment (Fig. 4G, H). Therefore, the effect of resveratrol to inhibit apoptosis and promote autophagy is mediated by dissociation of Beclin-1/Bcl-2 complex in AMPK/JNK1-dependent pathway.

### Protective effect of resveratrol on osteoporosis in ovariectomized rats

**Fig. 5:**
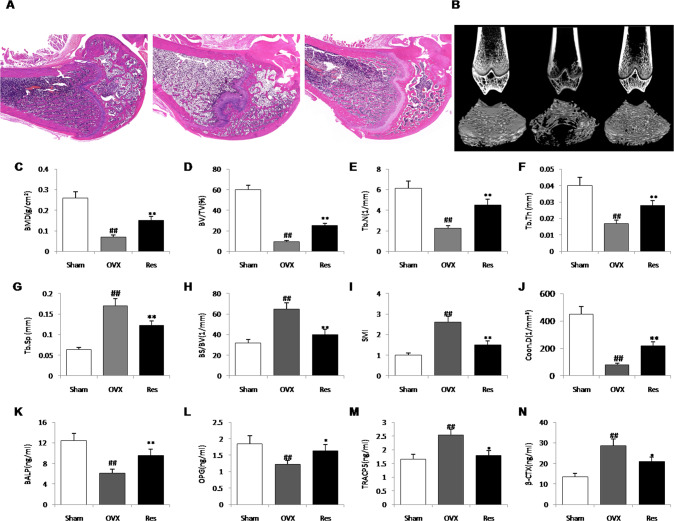
Protective effect of resveratrol on osteoporosis in ovariectomized rats. **A** The hematoxylin-eosin staining in distal femur among groups. **B** The 2D images of sagittal and transverse section. **C**–**J** The trabecula parameters of cancellous bone in the proximal tibial metaphysis static parameters: BMD bone mineral density, BV/TV bone volume/tissue volume, Tb.N trabecular number, Tb.Th trabecular thickness, Tb.Sp trabecular separation, BS/BV Bone surface/bone volume, SMI structure model index, and Conn.D connectivity Density. **K**–**N** serum bone alkaline phosphatase (BALP), osteoprotegerin (OPG), tartrate resistant acid phosphatase (TRACP-5b), and β-cross-linked c-telopeptide of type I collagen (β-CTX), *n* = 5, ^##^*P* < 0.01 vs. Sham group; *P < 0.05 vs. OVX group and **P < 0.01 vs. OVX group.

The HE staining showed that trabeculae significantly decreased and thinned in OVX group compared with the sham group, while resveratrol effectively reversed the alterations by a high number of trabecular bone and a decrease in trabecular bone separation (Fig. 5A). Consistent with the results of HE staining, micro-CT data showed that ovariectomy reduced caused a significant trabecular bone loss and deteriorated bone microarchitecture, which was indicated through the decreases of indicators such as BMD, BS/BV, BV/TV, Conn.D, SMI Tb.N, Tb.Sp and Tb.Th, compared to the normal rats. However, the administration of ovariectomized rats with 10 mg/kg resveratrol significantly reversed these bone indicators and enhanced the microstructural features of trabecular bones (Fig. 5B-J). In addition, serum levels of BALP and osteocalcin, bone formation markers, and Tracp 5b and β-CTX, bone resorption markers, were detected for assessing the turnover of the bones. BALP and osteocalcin levels were lower, but β-CTX and TRACP-5b levels were obviously higher in the OVX rats than those in the Sham rats (*P* < 0.01). The increased levels of BALP and osteocalcin and decreased levels of Tracp 5b and β-CTX were found in resveratrol-treated rats compared with those in OVX rats (*P* < 0.01) (Fig. 5K-N).

### Effects of resveratrol on osteocytes redox balancein in ovariectomized rats

**Fig. 6:**
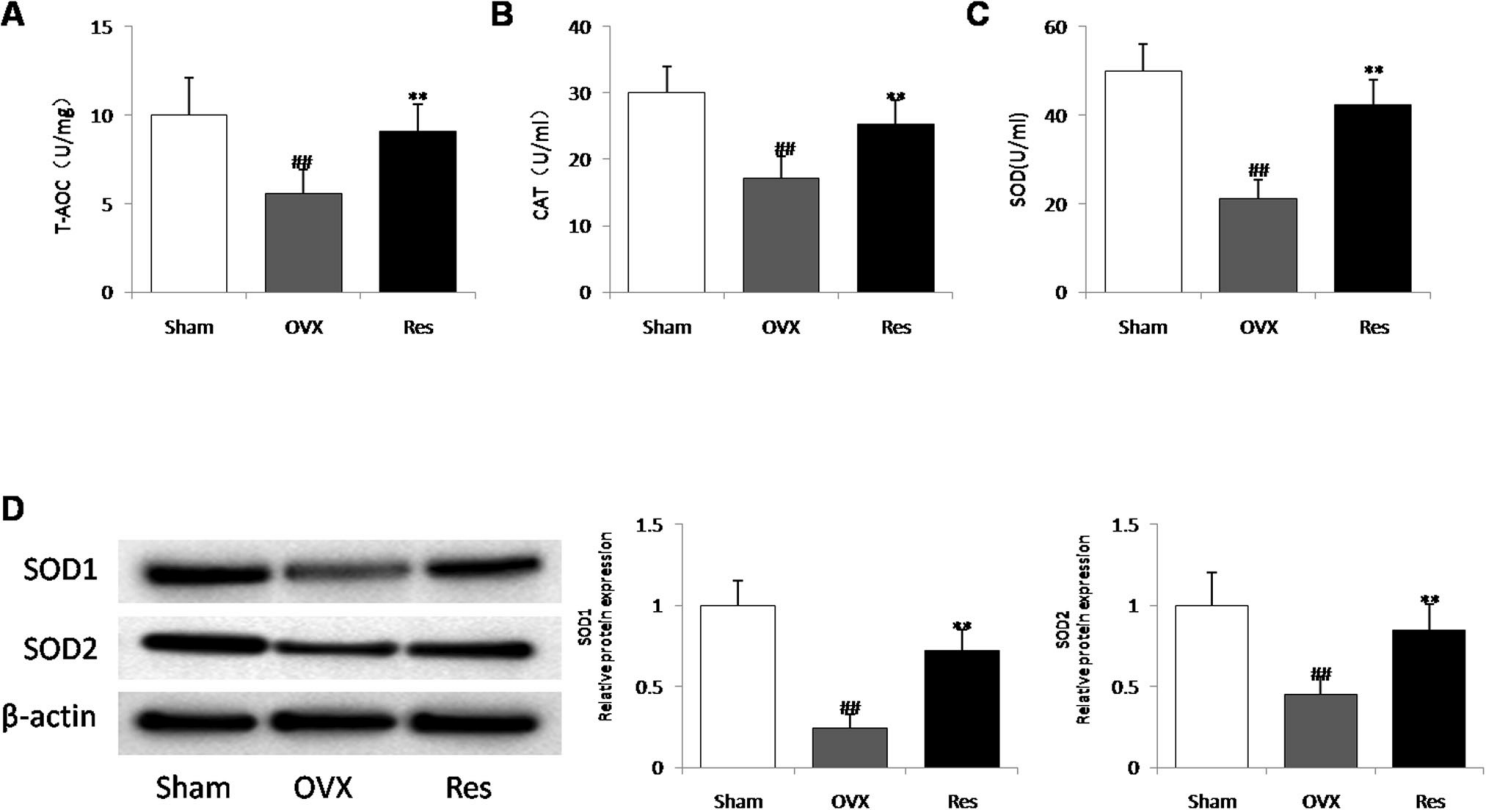
Effects of resveratrol on oxidative stress status in the ovariectomized model. **A**–**C** total antioxidant capacity (T-AOC) (**A**), catalase (CAT) (**B**), and superoxide dismutase (SOD) (**C**) activity in proximal tibias. (**D**) The protein expression levels of SOD1 and SOD2 were detected by western blot. Data were the means ± SD (*n* = 6 for each group). ^##^*P* < 0.01 *vs*. Sham group; **P < 0.01 vs. OVX group.

To determine whether postmenopausal prevention of resveratrol is related to a low oxidative stress of osteocytes, the activity and expression of antioxidant enzymes and ROS were examined in cortical bone of proximal tibias. Our results showed that ROS levels were obviously elevated, whereas superoxide dismutase (SOD) activity, catalase (CAT), total antioxidant capacity (tAOC), SOD1/SOD2 expression levels were markedly decreased in OVX group compared to sham group, but resveratrol treatment restored the changes of the above indicators (Fig. 6A-D). These results revealed that estrogen deficiency induced oxidative stress in bone tissue, and resveratrol administration reduced the oxidative stress of osteocytes in OVX rats.

### Resveratrol administration inhibited apoptosis and induced autophagy in the osteocytes of ovariectomized rats

**Fig. 7:**
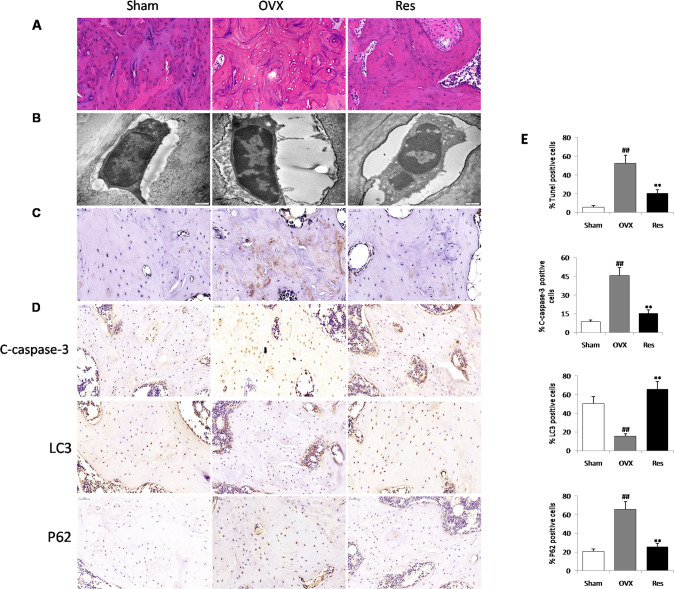
Resveratrol administration induced autophagy and inhibited apoptosis of osteocyte in ovariectomized rats. **A** Several osteocytes inside the lacunae are observed by hematoxylin-eosin staining. **B** Ultrastructural architecture of osteocytes in the proximal tibia. **C** The percentage of apoptotic osteocytes was assessed by Tunel staining in the proximal tibia. **D** Osteocyte-enriched bone sections were analyzed by immunohistochemistry for Cleaved caspase-3, LC3, P62. **E** Histomorphometric quantifications of the osteocytes number, ^##^*P* < 0.01 vs. Sham group; **P < 0.01 vs. OVX group.

HE staining results showed that the amount of osteocytes was remarkably decreased (*P* < 0.05) in OVX rats compared with sham rats, and the number of osteocytes increased significantly after resveratrol treatment (Fig. 7A). The ultrastructural analysis revealed osteocytes exhibited scarce cytoplasm with few organelles, irregular shape of nucleus and nearly all nuclei filled with condensed chromatin in the OVX rats. Resveratrol treatment obviously improved the pathological morphology of osteocytes (Fig. 7B). Tunel staining results showed that an obvious increase in Tunel-positive osteocytes in OVX rats compared with sham rats, whereas resveratrol could attenuate Tunel-positive osteocytes in the proximal tibia (Fig. 7C). Meanwhile, immunohistochemical staining results demonstrated that the numbers of caspase-3-positive and P62-positive osteocytes were increased and the numbers of LC3 + osteocytes were decreased in the proximal tibia of OVX rats, and resveratrol administration prominently reduced the number of caspase-3-positive and P62-positive osteocytes, and enhanced the number of LC3 + osteocytes in tibia shafts (Fig. 7D, E). This indicates that resveratrol has protective effects on osteocytes in ovariectomized rats by inducing autophagy and inhibiting apoptosis.

## Discussion

Osteoporosis is characterized by bone fragility and increased vulnerability to fractures due to a decrease in bone density and micro-architectural deterioration of bone tissues. Primary pharmacological strategies targeting osteoblasts and osteoclasts for the treatment of osteoporosis, including hormone replacement therapy (HRT), and alendronate therapies may not be recommended for long-term usage due to adverse side-effects [31]. Therefore, agents that simultaneously stimulate osteocytes activity and functions are needed. Numerous clinical trials and animal studies have shown that resveratrol can improve bone quality, regulate bone remodeling and delay osteoporosis [32], however, the exact mechanism involved has not been fully elucidated. In this work, the efficacy and mechanism of resveratrol on osteocytes were investigated using oxidative stress cell model and a rat model of osteoporosis. In this study, we found that resveratrol can suppress postmenopausal osteoporosis by regulating the switch between autophagy and apoptosis mechanism through activating AMPK and JNK1 pathway, and thereby protecting osteocytes from oxidative stress.

Osteocytes, as the most abundant cells in mammalian bone and regulate bone metabolism in coordination with osteoblasts and osteoclasts, play critical roles in maintaining bone homeostasis by acting as orchestrators and mechanosensors during the bone remodeling processes [33, 34]. Because osteocytes are long-lived cells residing within the bone matrix, they may be more susceptible to hypoxic conditions, ROS accumulation and nutrient deprivation [35].

Estrogen deficiency can increase oxidative damage and reduce antioxidant enzyme activity, which are the proximal culprits of osteoporosis [8, 36]. In this experiment, we established a hydrogen peroxide (H_2_O_2_) - mediated oxidative stress model of osteocytic cell lines in vitro. Our present study showed H_2_O_2_ stimulation induced excessive aggregation of reactive oxygen species in MLO-Y4 cells. Owing to the antioxidant effects, resveratrol ameliorated oxidative stress by reducing the levels of ROS. These excess free radicals may damage the cytomembrane and mitochondria, thus resulting in cell apoptosis [37]. Moreover, we also found that H_2_O_2_ induced apoptosis of MLO-Y4 cells, which was consistent with previous reports [38], and resveratrol suppressed the pathways of mitochondrial apoptosis by upregulating Bcl-2 expression and downregulating caspase-3 and Bax expression. Autophagy is not only important for the removal of damaged proteins and organelles, but also involved in the recycling of cellular components for maintaining energy balance [39]. Since osteocytes have long life-span and live under low-oxygen and -nutrient conditions, autophagy plays a key role in the survival of osteocytes [40]. Suppression of autophagy leads to the accumulation of damaged mitochondria which produce elevated levels of reactive oxygen species [41–43]. Furthermore, autophagy can protect against cell apoptosis [44]. Previous studies reported that autophagy was suppressed in response to chronic oxidative stress [45]. Our results demonstrated that prolonged exposure to oxidative stress could induce obvious autophagy inhibition in MLO-Y4 cells, and resveratrol can induce a beneficial autophagic process to protect the MLO-Y4 cells against H_2_O_2_-induced damage. Meanwhile, our results demonstrated that resveratrol could prevent MLO-Y4 cells from H_2_O_2_-triggered apoptosis by enhancing autophagic activities.

AMPK is a sensor of cellular energy status that plays a crucial role in the maintenance of energy homeostasis [46]. Moreover, activation of AMPK is involved in determining multiple cellular processes, including protein synthesis, cell growth, autophagy and apoptosis [47–52]. Previous studies have revealed that activation of AMPK stimulated JNK1–Bcl-2 signaling and disrupted the Beclin-1/Bcl-2 complex [53]. Bcl-2 / Beclin-1 complex plays a “switch” role in the development of autophagy and apoptosis. Beclin-1 may interact with Bcl-2 [54], and such interaction through BH3 domain suppresses Beclin-1-dependent autophagy by sequestering it away from class 3 PI3K [27, 28]. Our present study revealed that resveratrol activated AMPK/JNK1 pathways to dissociate Beclin-1/Bcl-2 complex. The free Beclin-1 binds to class 3 PI3K to generate a kinase complex and promote autophagy, while the free Bcl-2 can bind to pro-apoptotic molecules to suppress apoptosis [55]. Therefore, the current study suggested that resveratrol activated AMPK/JNK1, and thereby dissociating the Bcl-2/Beclin-1 complex, which may be an important mechanism of restoring autophagy and preventing apoptosis in response to oxidative stress.

The ovariectomized rat is a widely employed animal model for studying estrogen-deficiency in postmenopausal patients [56]. To understand the mechanisms underlying the in vivo effect of resveratrol on osteocyte of postmenopausal osteoporosis, estrogen-deficient rats were subjected to ovariectomy to establish an OVX model. First, the effect of supplementation of resveratrol on bone turnover parameters in OVX rats was assessed. Histological and micro-CT analyses revealed that resveratrol could positively affect trabecular bone microarchitecture, bone mineral density. Similarly, biochemical results confirmed that resveratrol decreased the serum levels of TRACP-5b and β-CTX, and a marked increase in the serum BALP and OPG in rats, thus indicating a dual therapeutic effect on stimulating bone formation and suppressing bone resorption following treatment with resveratrol. Consistent with the results of in vitro experiments, resveratrol administration for 12 weeks reversed, in part, OVX-induced reduction of antioxidative biomarkers in osteocyte. In addition, in accordance with previous study [57], the decreased number of osteocytes and the increased levels of TUNEL-positive osteocytes and cleaved caspases-3, but the reduced expression of beclin-1 and a significant increase of p62 were found in the proximal tibia of OVX groups rats, and resveratrol treatment effectively inhibit apoptosis and enhance autophagy of osteocyte in OVX rats. However, contrary to our findings, estrogen deficiency and replacement therapy could induce and suppress autophagic processes in the osteocytes of ovariectomized rats [58]. The reason for the contradictory conclusion is that autophagy modulation has differential effects on various cells, even in the same cell lineage over time [13]. The latter experiment was done 45 days after ovariectomy, while our experiment time was 12 weeks after modeling. These divergent results may also be attributed to the fact that osteocyte can undergo different autophagic processes depending on estrogen levels.

**Fig. 8:**
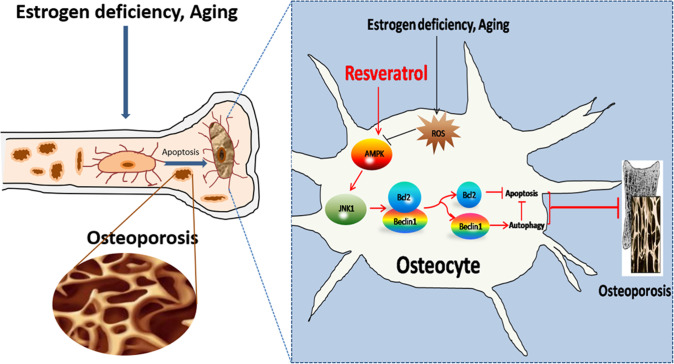
Schematic diagram for the molecular regulation of resveratrol against ovariectomized osteoporosis with osteocytes as target. Resveratrol protects osteocytes by regulating the switch between autophagy and apoptotic machinery under estrogen deficiency conditions, which is attributed by AMPK/JNK-mediated dissociation of Beclin-1-Bcl-2. ROS reactive oxygen species, AMPK Adenosine 5‘-monophosphate (AMP)-activated protein kinase, JNK1 c-Jun N-terminal kinase1.

In summary, our findings demonstrate that resveratrol prevents ovariectomy osteoporosis by inhibiting osteocytes apoptosis through promoting autophagy in rats. This effect is attributed by AMPK/JNK1-mediated disassociation of Beclin-1/Bcl-2 complexes (Fig. 8). Although the accurate switch between apoptotic and autophagic machinery in osteocytes needs to be further investigated in the future, and other signal pathways may also be involved in the protective effect of resveratrol on osteocyte autophagy activation and apoptosis inhibition, our results provide new evidence for the protective roles of resveratrol in postmenopausal osteoporosis.

## Materials and methods

### Cell culture and treatment

MLO-Y4 cells were purchased from iCell Bioscience Inc (Shanghai, China; Product No.: iCell-m037), grown in 0.15 mg/ml rat tail collagen type I-coated culture plates. α-MEM containing 2.5% bovine calf serum and 2.5% fetal bovine serum was added, followed by incubation at 37 °C in a 5% CO_2_ incubator [59]. In this experiment, the chronic stress state was used to simulate the physiological state of human body after estrogen deficiency. For chronic oxidative stress studies, MLO-Y4 cells were exposed to 0, 30, 60, or 120 μM H_2_O_2_ with or without resveratrol 25, 50, 100 μM (Sangon Biological Engineering Technology Company, Shanghai, China) as indicated daily for 14 d. The cells were pre-treated with 10 μM SP600125, 20 μM compound C (CC) and 10 μM 3-methyladenine 30 min before exposure to H_2_O_2_.

### Cell viability and oxidative stress assay

Osteocyte viability was evaluated by CCK8 assays. The cells (1 × 10^3^ cells/ml) were grown in 96-well plates, and the cell proliferation activity was measured at 24, 48, and 72 hr after inoculation using CCK8 (Dojindo, Kumamoto, Japan) with the accordance of manufacturer’s instruction. The absorbance values at OD450 were recorded using a microplate spectrophotometer (Thermo Labsystems, Vantaa, Finland). Oxidative stress was assessed by detecting malondialdehyde (MDA) and ROS levels according to the manufacturer′ instructions. ROS levels were assessed with DCHF-DA probes.

### FITC-annexin V and PI staining

Osteocyte apoptosis was detected with FITC-Annexin V and PI staining by following the kit’s protocols (Clontech, CA). After growing in a 35-mm plate at 37 °C overnight, MLO-Y4 cells were trypsinized and incubated with PI solution (5 μl) and FITC-Annexin (5 μl) in the dark for 15 min. MLO-Y4 cells apoptosis rates were analyzed by FACS.

### Immunofluorescence confocal microscopy

The expression of LC3 in MLO-Y4 cells was determined via immunofluorescence. MLO-Y4 cells were grown in a 24-well plate on slides. After treatment, the cells were fixed in paraformaldehyde (4%), and permeabilized with Triton X-100 (0.1%). The cells were incubated with anti-LC3 primary antibody (Code n. sc-135836, Santa Cruz Biotechnology, USA); and then probed with fluorophore-conjugated secondary antibodies and DAPI. The confocal imaging was analyzed using an FV1000 Olympus laser scanning confocal microscope (Olympus, Japan).

### Immunoprecipitation and immunoblotting

MLO-Y4 cells were homogenized in lysis buffer, and protein concentration was quantified using the Bradford assay. After separation through 15% SDS-PAGE and transferred onto PVDF membranes, cell lysate was subjected to immunoprecipitation with specific antibodies for 1 h at RT, followed by immunoblotting according to the kit’s protocols (Roche, Basel, Switzerland). The primary antibodies used for immunostaining were: rabbit anti-JNK1 (1:1000, Abcam, ab199380), rabbit anti-p-JNK1 ab47337, rabbit anti-AMPK (1:1000, Abcam, ab32382), rabbit anti-p-AMPK (1:1000, Abcam, ab133448), rabbit anti-Beclin 1(1:1000, Abcam, ab207612), rabbit anti-Bcl-2 (1:1000, Abcam, ab182858), rabbit anti-LC3 (1:1000, Abcam, ab128025), rabbit anti-Cleaved Caspase-3 (1:1,000, Abcam, ab32042), rabbit anti-Cleaved PARP (1:1000, Abcam, ab32064), rabbit anti-SOD1 (1:1000, Abcam, ab179843), rabbit anti-SOD2 (1:1000, Abcam, ab74231), rabbit anti-Actin (1:3000, Abcam, ab8227). The secondary antibodies used for immunostaining were: mouse HRP (1:2000, Abcam, ab6728), rabbit HRP (1:2000, Abcam, ab6721).

### Animals and treatments

Female SD rats (6 months old) in this experiment were supplied by the Laboratory Animal Center of Zhengzhou University. The rats were raised in a 12-h day and night cycle with free water intake at 23 °C, and then andomized into 3 groups (*n* = 8 per group): ovariectomized model group (OVX), sham surgery with intact ovaries group (Sham), and resveratrol group (Res). The sample size was determined by considering statistical considerations and ethical concerns. All animals that participate in the experiment were included for analysis, unless animal death. The rats in OVX and Res groups underwent bilateral ovariectomy (OVX) under anesthesia with an intraperitoneal injection of 0.3% sodium pentobarbita per 0.1–0.2 ml/10 g of body weight. An additional 8 rats underwent sham surgery. Two weeks after ovariectomy, rats in OVX and Res groups were received respectively resveratrol solution at 10 mg/kg body weight by daily intraperitoneal injection or saline. After 12 weeks of treatment, the rats were sacrificed, blood, proximal tibias, distal femurs, lumbar vertebrae were collected. The study procedures were approved by the Experimental Animal Ethics Committee at orthopedics hospital of Henan province (Animal Protocol Approval No.: 201803010), and conducted in accordance with ethical requirements and ARRIVE guidelines.

### Serum markers of bone formation and resorption

Blood specimens were withdrawn from the abdominal aorta after 8 weeks of treatment. Separation of the serum was accomplished by centrifugation (3000 rpm, 15 min, 4 °C). The levels of serum TRACP-5b, β-CTX, OPG, and BALP were measured with ELISA kits by following the kit’s protocols.

### Measurements of SOD, CAT, and tAOC

The proximal tibial bone tissue (100 mg) was homogenized with the aid of dry ice. As cortical bone has a more concentration of osteocytes, it would be more suitable to collect homogenates only from cortical bone compartment. After centrifugation (1000 g, 4 °C, 10 min), the levels of SOD, CAT, and tAOC in supernatant were determined with commercial kits (Beyotime, Jiangsu, China) by following the kit’s protocols. The protein levels of SOD1 and SOD2 were assessed by immunoblotting.

### Micro CT analysis

Micro CT (μCT 80, Scanco Medical, Zurich, Switzerland) was used to evaluate the microstructure of trabeculae at the epiphysis of femoral shaft. Bone trabeculae are abundant in the distal femur. Each selected sample was scanned from the distal to the proximal at a resolution of 16 μm. The region of interest (ROI) of trabecular bone started from 0.15 mm proximal to distal growth plate, and extended proximally for 0.4 mm. The parameters in the ROI, such as bone volume over total volume (BV/TV), bone mineral density (BMD), trabecular bone number (Tb.N), trabecular bone thickness (Tb.Th), and trabecular bone separation (Tb.Sp), were automatically calculated using the micro-CT system’s software, and the analysis threshold was 245.

### Histological analysis

The proximal tibias were fixed in paraformaldehyde (4%) for 48 hr, decalcified in 15% EDTA at room temperature for 2 weeks, and paraffin-embedded. Then, 4 μm slices were coronally sectioned, followed by hematoxylin-eosin (HE) staining. The stained proximal tibias were examined using a light microscope (Nikon Eclipse 80i, Tokyo, Japan).

### TEM assay

The proximal tibial tissues were fixed in glutaraldehyde (2%) for 24 hr, followed by demineralization in EDTA (5%) for 14 days. After cutting into 1mm^3^, the specimens were postfixed in 1% OsO4 and embedded with epoxy resin. Next, the specimens were sectioned at 80-nm sections, followed by uranyl acetate and lead citrate staining. Lastly, the ultrathin slides were subjected to TEM analysis (Phillips CM-80, Netherlands).

### Tunel assay

Tunel assay was conducted with ApopTag Plus Peroxidase in situ apoptosis detection kit by following the kit’s protocol (KeyGEN Biotech, Nanjing, China). Briefly, the sections of proximal tibias were dewaxed and hydrated by routine methods. After incubation with Tunel reaction mixture at 37˚C for 1 h, the sections were stained with DAB substrate at room temperature for 10 min. Apoptotic cells were observed using a fluorescence microscope (CX41RF; Olympus Corporation, Tokyo, Japan).

### Immunohistochemical staining

The paraffin-embedded femur tissues were sectioned at 3-μm thickness. After incubation with cleaved caspase-3 antibody (Cell Signalling Technology, Boston, MA, 1:100), LC3 antibody (Abcam, Cambridge, MA, 1: 200) and P62 antibody (Santa Cruz Biotechnology, CA, 1: 100) at 4 °C overnight, then incubated in HRP-labeled goat anti-rabbit secondary antibody (Beyotime Institute of Biotechnology, Inc., Jiangsu, China) for 30 min at 37 °C. After another 30-min incubation with Vectastain ABC-AP kit (Vector, CA), the specimens were rinsed and exposed to 3,3-diamino-benzidine (DAB) substrate for 3–10 min.

### Statistical analysis

Data were obtained from at least three separate experiments performed in triplicate. All values are shown as means ± standard deviations (SD). Statistical analysis was undertaken only for studies where each group size was at least *n* = 5. The difference in means between two groups was compared by two-tailed Student’s *t* test. One-way analysis of variance (ANOVA) and two-way ANOVA was performed for multiple comparisons. The variance was similar between the groups that were being statistically compared. All statistical tests were conducted with GraphPad Prism software, and *P* < 0.05 was deemed statistically significant.

### Blind statement

The investigator was blinded to the group allocation during the experiment and/or when assessing the outcome.

## Supplementary information

western blot original picture 1

western blot original picture 2

## Data Availability

All data generated or analyzed during this study are included in this published article.
