# Supplementary figures and images for: Resveratrol protects osteocytes against oxidative stress in ovariectomized rats through AMPK/JNK1-dependent pathway leading to promotion of autophagy and inhibition of apoptosis
Source: Cell Death Discov. 2023 Jan 21;9:16. doi: 10.1038/s41420-023-01331-2 (PMC9867734; doi:10.1038/s41420-023-01331-2)

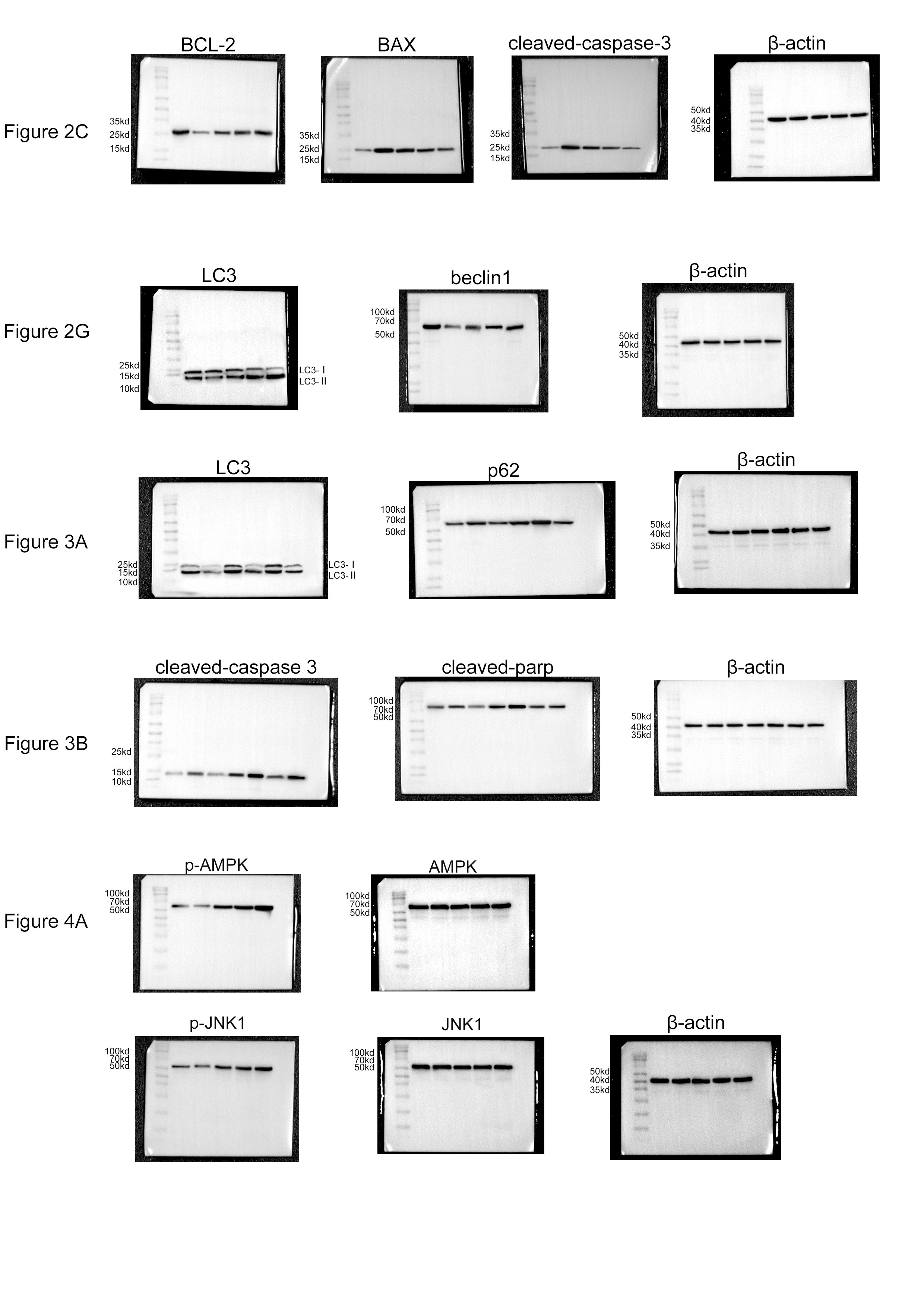

Supplement: Supplementary file 1 — western blot original picture 1 [file 41420_2023_1331_MOESM1_ESM.tif]

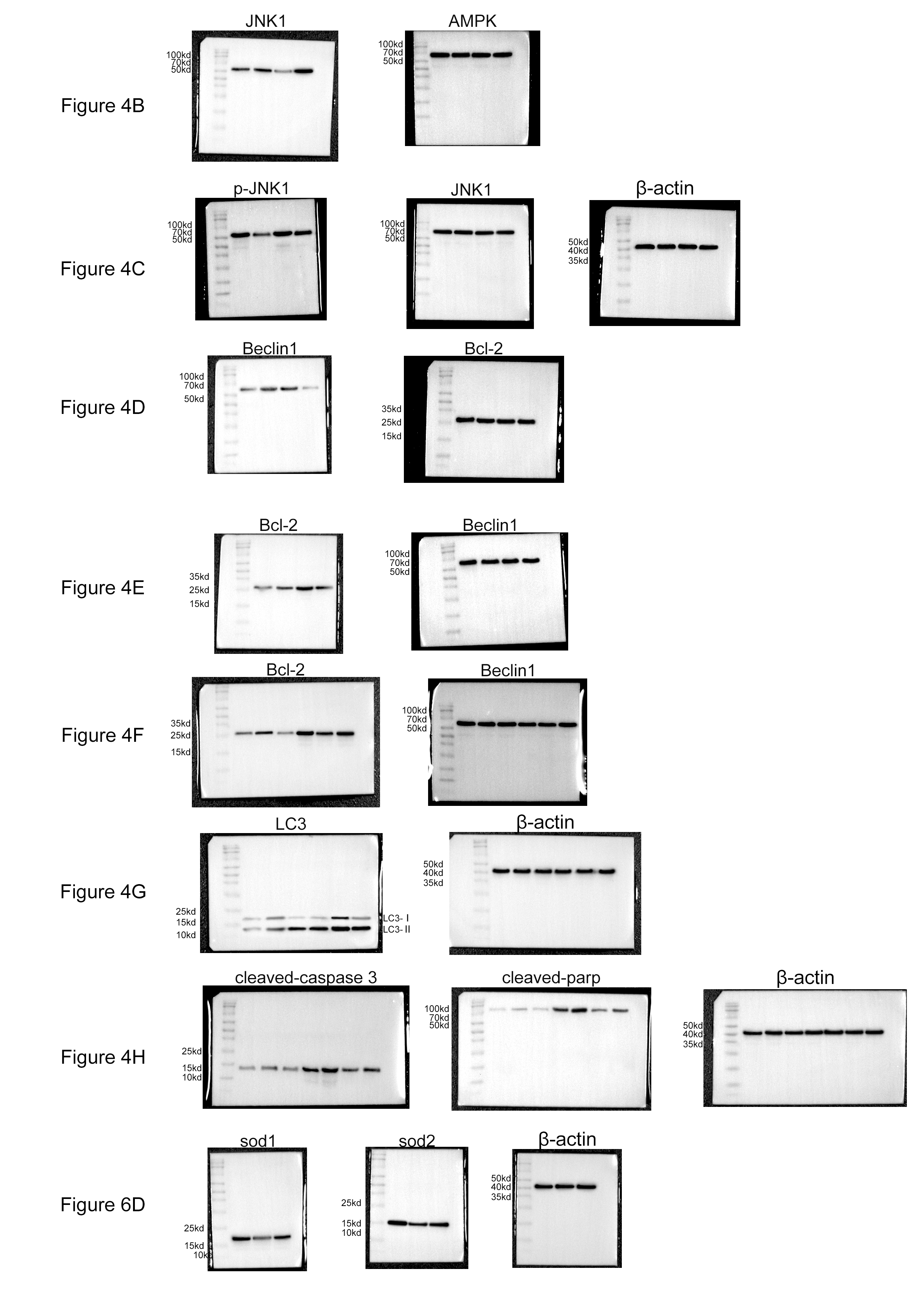

Supplement: Supplementary file 2 — western blot original picture 2 [file 41420_2023_1331_MOESM2_ESM.tif]
